# Large-Scale Clonal Analysis Reveals Unexpected Complexity in Surface Ectoderm Morphogenesis

**DOI:** 10.1371/journal.pone.0004353

**Published:** 2009-02-06

**Authors:** Anne-Cécile Petit, Jean-François Nicolas

**Affiliations:** Unité de Biologie moléculaire du Développement, Institut Pasteur, Paris, France; The University of Hong Kong, China

## Abstract

**Background:**

Understanding the series of morphogenetic processes that underlie the making of embryo structures is a highly topical issue in developmental biology, essential for interpreting the massive molecular data currently available. In mouse embryo, long-term *in vivo* analysis of cell behaviours and movements is difficult because of the development *in utero* and the impossibility of long-term culture.

**Methodology/Principal Findings:**

We improved and combined two genetic methods of clonal analysis that together make practicable large-scale production of labelled clones. Using these methods we performed a clonal analysis of surface ectoderm (SE), a poorly understood structure, for a period that includes gastrulation and the establishment of the body plan. We show that SE formation starts with the definition at early gastrulation of a pool of founder cells that is already dorso-ventrally organized. This pool is then regionalized antero-posteriorly into three pools giving rise to head, trunk and tail. Each pool uses its own combination of cell rearrangements and mode of proliferation for elongation, despite a common clonal strategy that consists in disposing along the antero-posterior axis precursors of dorso-ventrally-oriented stripes of cells.

**Conclusions/Significance:**

We propose that these series of morphogenetic processes are organized temporally and spatially in a posterior zone of the embryo crucial for elongation. The variety of cell behaviours used by SE precursor cells indicates that these precursors are not equivalent, regardless of a common clonal origin and a common clonal strategy. Another major result is the finding that there are founder cells that contribute only to the head and tail. This surprising observation together with others can be integrated with ideas about the origin of axial tissues in bilaterians.

## Introduction

In complex development, cells of the embryo are rearranged by cell movement and other cell behaviours [1], [2] that shape the embryo and generate structures. Amniotes development occurs during periods of intense cell proliferation. As a result the signals to which cells are exposed change, with two consequences. It increases considerably the repertoire of combinatorial signals that the embryo can exploit, an evolutionarily favourable outcome. It generates the need for tight control of cell rearrangement and changes in shape, imposing major constraints on developmental processes [3]. How cell behaviour is exploited for morphogenesis and coupled to cell specification are major issues in developmental biology and are also of importance for the understanding of cellular operations evolution and their genetic control in animal groups [4], [5], [6].

Analysis of the contribution of cell rearrangement and movement in mouse morphogenesis by following the embryo *in vivo* is difficult because of its inaccessibility and the impossibility of long-term culture [7], [8]. Genetic methods of clonal analysis [9], [10], [11], [12] present an alternative that can provide information about cell proliferation, mode of growth, cell rearrangement and other aspects of cell behaviour [13], [14], [15], [16], [17], [18], [19], [20], [21].

We improved two clonal analysis methods: the LaacZ method of random induction of labelling [11], [14], [19], [22], [23] that has been modified to make all cell types visualizable [24]; a method of genetic induced cell labelling [20], abbreviated GICL in this article, adapted from genetic induced fate mapping (GIFM) techniques [25], that allows temporal induction of the labelling and has been modified to permit the labelling of any cell in the early embryo, all cell types being also visualizable. Combined together, these two methods permit large-scale production of labelling.

We present an analysis, using these methods, of the formation of surface ectoderm (SE) from E6.5 to E14.5, a period that includes gastrulation and the establishment of most structures of the organism. We report that SE, a simple 2D monolayer epithelial structure, shows non-random cell behaviours, namely that SE formation and elongation involve different combinations of cell rearrangement and modes of cell proliferation according to position along the axis. Our results suggest that the posterior zone in the embryo is crucial for SE elongation; cell proliferation and cell rearrangement are temporally and spatially organized in this zone. Another finding is that there is an early common pool of precursors restricted to the head and the posterior part of the embryo. This puzzling observation is consistent with ideas about the origin of the axial tissues in bilaterians.

## Results

### The global LaacZ method and the SE LaacZ library

The LaacZ method has been made ubiquitous by introducing a *LaacZ* reporter gene into the ROSA26 locus [24]. The ROSA26 promoter confers ubiquitous expression of LacZ [26]. The 1117bp duplication in the coding sequence of the LaaZ gene generates multiple in-frame stop codons. As a consequence, the LaacZ gene encodes a non-functional β-galactosidase and non-sense mediated decay is induced [27]. A functional LacZ gene can be restored by spontaneous intragenic homologous recombination within the duplicated region. The recombined LacZ is then transmitted to all descendants of the modified cell. The resulting clone is detectable by β-galactosidase histochemical staining [22], [28]. The ROSA26LaacZ method allows visualization of any clonally-related cell [24]; it therefore ensures that no area of the structure of interest is excluded from the analysis.

A SE LaacZ library has been produced. It contains 4248 E14.5 embryos. To validate the library, a sample of 97 embryos has been screened for determining the number of embryos lacking SE labelling. 42 (44%) such negative embryos were found. From this number, the expected number of labellings corresponding to N recombination events (from 1 to 4) was calculated using the fluctuation test of Luria and Delbrück (see Materials and Methods). 35 (36%) embryos are expected to show clonal labelling (N = 1); 15 (15%), two recombination events (N = 2) and only 4 (4%), three recombination events (Table 1, left column). Owing to the independent nature of the recombination events in both time and space, most of the double or triple recombination events correspond to situations readily recognizable by the size and spatial disposition of labelled clusters. Indeed most double events involve a small second clone and most non-clonally related clusters are scattered in SE. In addition these composite patterns are expected to be non-reproducible. We then applied these three criteria (size, spatial disposition and pattern non-reproducibility) to the description of the 97 embryos. The observed numbers of labelling possibly corresponding to N events (Table 1 right column) strikingly corresponded to those calculated using the fluctuation test (Table 1 left column). This suggests that the above reasoning is correct.

**Table 1:** The LaacZ library: frequency of embryos with N events of recombination.

N	No of embryos with N recombination events expected	No of embryos with N clones observed
0		42 (43%)
1	35 (36%)	30 (31%)
2	15 (15%)	15 (15%)
3	4 (4%)	9 (9%)
4	1 (1%)	1 (1%)
		97

The validity of the LaacZ library was assessed by determining the number of embryos totally lacking SE labelling on a sample of 97 embryos. From this number (42) the number of embryos presenting N events of recombination (from 1 to 4, left column) was calculated using the fluctuation test of Luria and Delbrück (see Materials and Methods). The right column reports the observed numbers of embryos possibly corresponding to N events (see text for details).

The characteristics of the whole library are summarized in Table 2. The number of medium (50 to 100 cells) and large labellings (more than 100 cells) on which this study is based are small (4% to 0.1%). In consequence the probability that they derive from two recombination events (see Materials and Methods) is negligible and thus cannot impact our analysis. For instance, the probability that a clone composed of 200 to 400 cells arises from two clones composed of 100 to 200 cells is equal to 1.9.10^−4^ (2 embryos for 10 000 observed) and the probability that it arises from 4 clones of 50 to 100 cells is 2.5.10^−6^ (3 embryos for one million observed). Similarly the probability that a clone composed of 400 to 800 cells arises from two clones composed of 200 to 400 cells is equal to 1.2.10^−5^ (1 embryo in 100 000 observed).

**Table 2:** The LaacZ library: size composition of the clones.

No of cells	No of embryos with labelling composed of n cells	No of embryos with labelling composed of n cells
n	Entire library	Sample of 97 embryos
>800	0.1% (6)	0% (0)
400<n<800	0.4% (16)	0% (0)
200<n<400	0.4% (15)	1% (1)
100<n<200	1.4% (59)	3% (3)
50<n<100	4% (165)	7% (7)
20<n<50	nd	12% (12)
10<n<20	nd	21% (20)
<10	nd	51% (49)
Total no of embryos	4248	97

SE clones are classified according to the number of cells they contain. The frequency of large clones was determined in the entire library. The frequency of small clones was determined with a sample of 97 embryos.

### GICL and the SE lox-LacZ library

The method of production of labelling whose birth date can be controlled is based on Cre recombinases [29], [30], the activity of which depends of a conformational change induced by 4-hydroxytamoxifen (4-OHT) [31]. This approach is generally combined with tissue-specific expression of the recombinase for somatic mutagenesis [32] and GIFM [25]. In GIFM, the aim is to induce genetic deletion in the highest number of cells at a given stage of development. In GICL the aim is to induce labelling in a single cell at a given stage of development. Here we report the first use of ubiquitous GICL to analyse SE, a large structure of the embryo.

Ubiquitous GICL presents four major specific constraints: 1) every single cell of the animal must express the recombinase, thus making possible the induction of clones in any structure and at any time during development; 2) the recombined reporter gene must be expressed in every descendant of the recombined cell, thus allowing the detection of labelled cell descendants at any selected stage of observation; to fulfil these two criteria, we used inducible ROSA26-driven Cre recombinases and a ROSA26 LacZ reporter gene [26]; 3) the conditional reporter line must be immune to spontaneous recombination that could activate the reporter gene in the absence of the Cre recombinase. ROSA26 LacZ reporter line (R26R) fulfils this criterion as no spontaneous recombination was ever found in R26R embryos and animals (E. Legué, unpublished data); 4) ideally, the inducible Cre recombinase must have no activity in absence of the inducer molecule; in practice, the spontaneous activity of the inducible Cre recombinase must be low enough to allow the distinction between induced clonal labellings and background linked to spontaneous labellings. It appeared that the ROSA26cre-ER^T^ line [33], that can be used to produce polyclonal labelling (see below), is improper for clonal labelling because of a significant level of spontaneous recombination. Another ROSA26 Cre line, CT2 (L. Grotewold and A. Smith, unpublished), in which a Cre-ER^T2^ gene has been introduced at the Nhe1 restriction site located 1 kb downstream of the usually used Xba1 restriction site, presents a frequency of spontaneous labelling compatible with the generation of a library of SE clones induced during gastrulation. Indeed the expected size of SE clones induced at E6.5 and observed at E14.5 is about 256 cells (2^8^, assuming a doubling time of 24 h) and in [CT2×R26R] embryos the frequency of spontaneous labelling of clones with 200 to 400 cells is only 1.3×10^−2^ (Table 3, first line). This allows inducing clones at a frequency fifteen time above that of the background (up to about 2×10^−1^). At this value, the frequency of double induction events is only 4×10^−2^ (see Materials and Methods), that is too unfrequent to have an incidence on our analyses.

**Table 3:** The lox-LacZ library of clones induced at E6.5 and observed at E14.5.

4-OHT dose	Total no of embryos	No of embryos with clones composed of more than 400 cells	No of embryos with clones composed of 200 to 400 cells
Non injected	144	2% (3)	1% (2)
0,44 µg.g^−1^ iv	161	8% (13)	9% (15)
		χ^2^ = 5,49 ; p = 0,019	χ^2^ = 9,08 ; p = 0,0026
0,33 µg.g^−1^ iv	126	12% (15)	17% (21)
		χ^2^ = 10,4 ; p = 0,012	χ^2^ = 22,7 ; p<0,0001

The 64 SE clones in the library are from several series of injection. The χ^2^ and Fisher's exact probability tests (χ^2^ corr) were used to compare the experimental groups with the control groups (first line).

A library of SE clones induced during gastrulation and referred to as the lox-LacZ library has been produced using 4-OHT concentrations yielding labelling frequencies between 1 and 2×10^−1^ (Table 3). To further increase the stringency of the library, we look for potential variability in the induction frequencies by comparing litters 2 by 2 using Fisher's exact test. The two extreme categories (the least labelled potentially not induced and the most labelled potentially too induced) were discarded. The statistical analysis of the final library (64 labelling among 287 embryos) that confirms its validity is reported in Table 3.

### Terminology

In this article, the following terminology [3], [9], [23], [34] is used. The ancestor cells of a structure are defined as any cell that will contribute at least some descendants to this structure and also to other structures in the embryo. The founder cells of a structure are defined as the first cells of a lineage whose contribution is restricted to that structure [34]. The extent of clone contribution to different structures and tissues of the embryo defines ancestral and founder clones. During coherent growth, sister cells remain close to each other, while dispersive growth results in widely separated sister cells. In addition growth can be either oriented or isotropic. The analysis of the spatial distribution of a clone informs about its mode of growth. In a library of clones, saturation is reached when the library has more than one example of any possible labelling pattern. At this stage, additional clones do not provide new information. The clonal complexity of a region is equal to the number of clones that contribute to this region. Clonal complexity can be used to detect mode of growth and territory of preferential growth in a structure [14]. For this kind of analysis, the structure of interest is divided in regions and contributions of the clones from a library at saturation to these regions are determined and compared.

### Cell behaviour during SE formation

**Figure 1:**
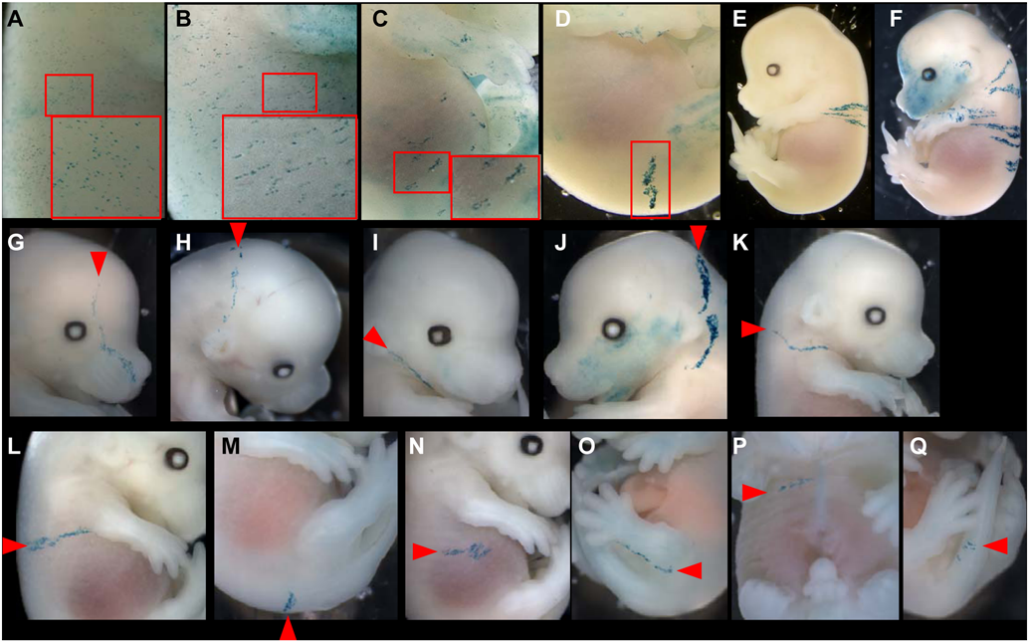
SE labelling induced from E6.5 to E13.5 reveals a single clonal strategy for all regions of the embryo. (A–C) [ROSAcre-ER^T^×R26R] and (D–F) [CT2×R26R] embryos. Pregnant mice injected with 4-OHT at E13.5 (A), E12.5 (B), E9.5 (C), E8.5 (D), E7.5 (E), E6.5 (F). Observation of E14.5 embryos. Growth is isotropic (A); dorso-ventrally oriented and coherent in (B) to (D). In (E) and (F), growth is dispersive and results in longitudinally dorso-ventrally oriented stripes. (G), (I), (K–Q) Examples of clones observed in LaacZ embryos; (H), (J) Examples of clones observed in lox-LacZ embryos induced at E6.5. (G)–(J) in head regions, (K) to (P) in the trunk and (Q) in the tail. Arrowheads indicate the most dorsal position to which the clones contribute.

To determine the cell behaviour involved in the formation of SE, polyclonal labellings were first induced between E8.5 and E13.5 and observed at E14.5 ([R26CreER^T^ xR26R] E6.5 to E13.5 libraries). After induction at E13.5, the labelling revealed coherent and isotropic groups of cells (Fig. 1A). Similar but more extensive labelling was observed in newborn mice. SE growth is therefore coherent and isotropic from E14 to post-natal or later stages, as expected [35]. After induction between E8.5 and E12.5, coherent stripes of cells oriented dorso-ventrally were labelled, and were larger when induction was earlier (Fig. 1B–D). Thus, SE growth between E9 and E14 is coherent and oriented.

Then clonal labellings were induced between E6.5 and E7.5 and observed at E14.5 ([CT2×R26R] E6.5 and E7.5 libraries, hereafter called the lox-LacZ libraries). Clonal induction between E6.5 and E7.5 resulted in groups of a few DV-oriented stripes distributed along the AP axis (Fig. 1E induced at E7.5 and F induced at E6.5). A period of cell dispersion along the longitudinal axis of the embryo therefore precedes the period of coherent and oriented growth along the DV axis. This period corresponds to the first stages of elongation of the embryo.

These findings suggest a clonal strategy involving a mechanism that distributes cells longitudinally, then a mechanism that arrests cell dispersion and produces oriented stripes, followed by a mechanism that shifts oriented growth to isotropic growth. The clonal signature of this strategy is the DV oriented stripes.

### The same clonal strategy is used in all regions of the embryo

We next investigated whether this clonal strategy is used in all regions of the SE. Therefore we searched for clones composed of a single DV stripe in the LaacZ and lox-LacZ libraries. Such clones were found in all regions of the head: the facial region (Fig. 1G), the encephalic region (Fig. 1H), the maxillary region (Fig. 1I) and the neck (Fig. 1J); the trunk, dorsal (Fig. 1K–M), lateral (Fig. 1N, O) or ventral (Fig. 1P), from the anterior limit of the forelimb (Fig. 1K) to the posterior limit of the hindlimb (Fig. 1O); and the tail (Fig. 1Q).

The presence of this clonal signature in all regions of the embryo suggests that the same cell behaviour is involved in all SE regions. We named the precursor cells of the DV-oriented stripes Precursor of DV-oriented Clonal Unit (P-DVCU) and the DV-oriented clonal unit DVCU. We next followed the clonal history of these precursors to determine how they are produced and positioned longitudinally and dorso-ventrally and whether they are governed by different modes of cell behaviour.

### A pool of SE founder cells already regionalized for its dorso-ventral contribution

We used the LaacZ library of E14.5 embryos to study how the pool of SE founder cells (see terminology) is formed.

**Figure 2:**
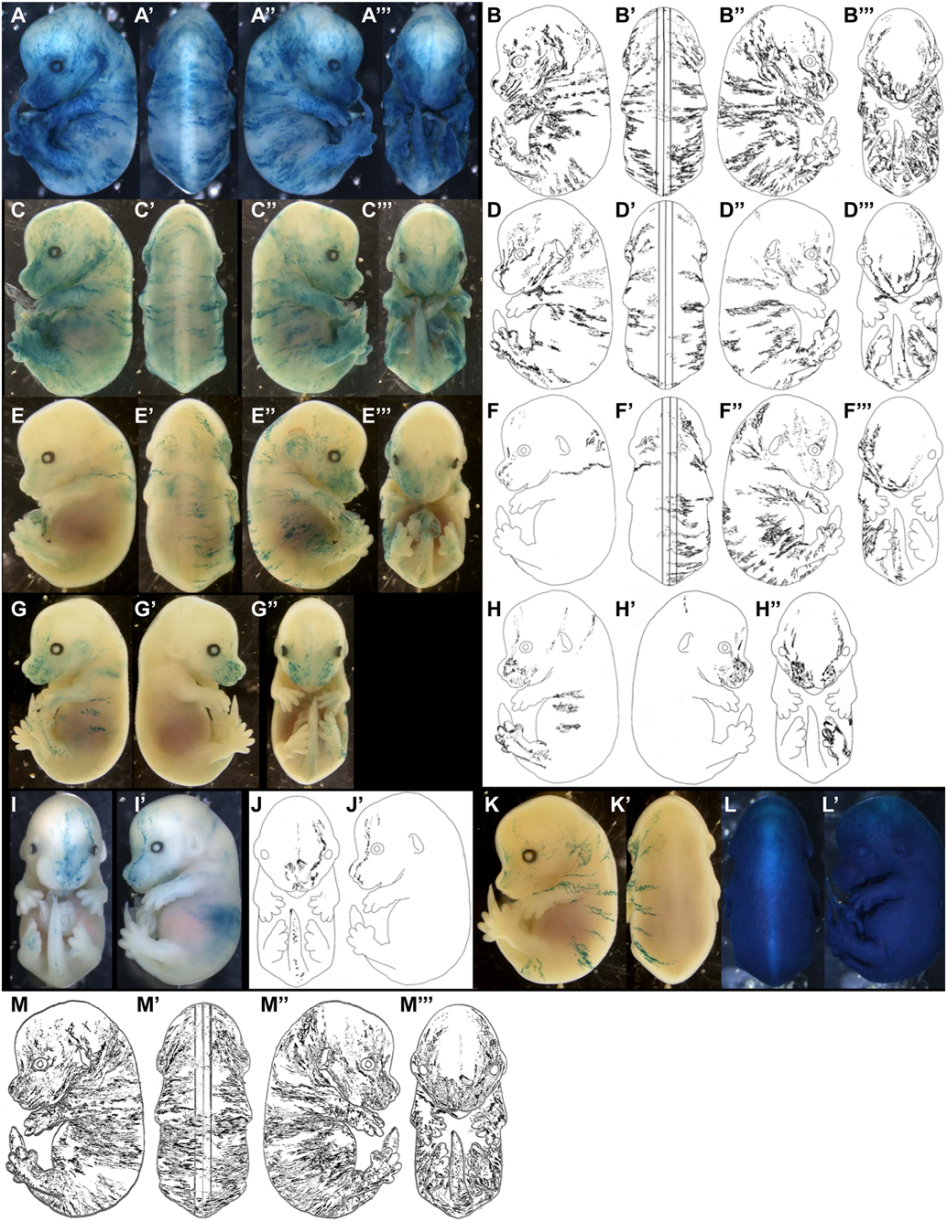
Ancestral and founder cells of the surface ectoderm. Examples of clones dispersed along the entire SE observed in E14.5 LaacZ embryos. (A)–(J′) non-SE-restricted clones classified from biggest to smallest. (K–K′) the biggest SE-restricted clone. (L–L′) the biggest non SE-restricted clone. (M–M′′′) Superimposition of (B–B′′′), (D–D′′′), (F–F′′′), (H–H′′′), and (J–J′′′); note the lack of labelling in the most dorsal region of SE delimited by the lines. (A), (C), (E), (G), (I) *in toto* X-gal staining. (B), (D), (F), (H), (J) drawings of their SE contribution.

As founder cells derived from ancestral cells (see terminology), we first searched for ancestral clones. Seven clones contributing to both SE and internal structures (Fig. 2A–J′ and L–L′) that is, labelled before the restriction of cells to the SE, were found. In six clones (Fig. 2L–L′), the labelling was mosaic in the SE, indicating that the SE is derived from several founder cells, and therefore that there are groups of clonally related P-DVCUs.

The clones with the largest total cell number (Fig. 2A–D and L) made the largest contributions to the SE compared to clones with fewer cells (Fig. 2E–J). The size of the clone correlates with its date of birth and therefore the earliest ancestral cells produce more SE founder cells than the later ones.

The labelling pattern in the large ancestral clones (Fig. 2A and C) included both sides and all regions of the SE; in the smaller ones (Fig. 2E–J) it was mostly restricted to only one side of the SE. The bilateral contribution of SE precursor cells is therefore restricted very early.

The clonal pattern of the embryo in Fig. 2G is restricted to part of the DV axis of the SE. The contribution of SE founder cells is therefore DV restricted. This restriction occurs before the establishment of the pool of founder cells (compare Fig. 2E–F and G–H).

Finally, all seven clones of ancestral cells exhibit an extensive AP labelling from the head to the tail. Therefore there is no obvious AP restriction of the ancestors and founder cells.

The labelling shown in Fig. 2K–K′ is the most extensive restricted to the SE, found in the LaacZ library. The clone exhibits all the characteristics described for the labelling of the ancestral cells of the SE: it is unilateral, extends along the whole AP axis and its contribution to the DV axis is restricted. It may correspond to the labelling of a SE founder cell.

To assess whether the whole SE can be produced from the founder cells descended from the seven ancestral clones, their contributions were superimposed on a single schematic representation of an E14.5 embryo (Fig. 2M–M′′′). Labelling was observed in all AP and DV regions, including those formed late such as the posterior regions. The region above the neural tube was under represented (Fig. 2M, the region delimited by the lines). It is therefore not necessary to invoke recruitment from another source for any regions of the embryo including the late-formed structures; the pool of SE founder cells is probably a closed pool from an early stage of embryogenesis.

These analyses showed that each cell of the initial pool of SE founder cells produces large groups of P-DVCUs. These P-DVCUs are not randomly distributed in the embryo; they are arranged longitudinally and their contribution is DV restricted. The most dorsal part of the SE is under-represented relative to all other regions. If it is produced from founders that are restricted along their DV axis, like the other founders, the corresponding pool is smaller. The ancestral cells of the SE are not equipotent. They can produce different numbers of organized founder cells whose properties are not equivalent. This reveals that the ancestral cells show a certain level of coherence and do not mix freely with the other cells of the embryo before their allocation to the SE.

### The initial pool of SE founder cells is regionalized into three between E6.5 and E7.5

**Figure 3:**
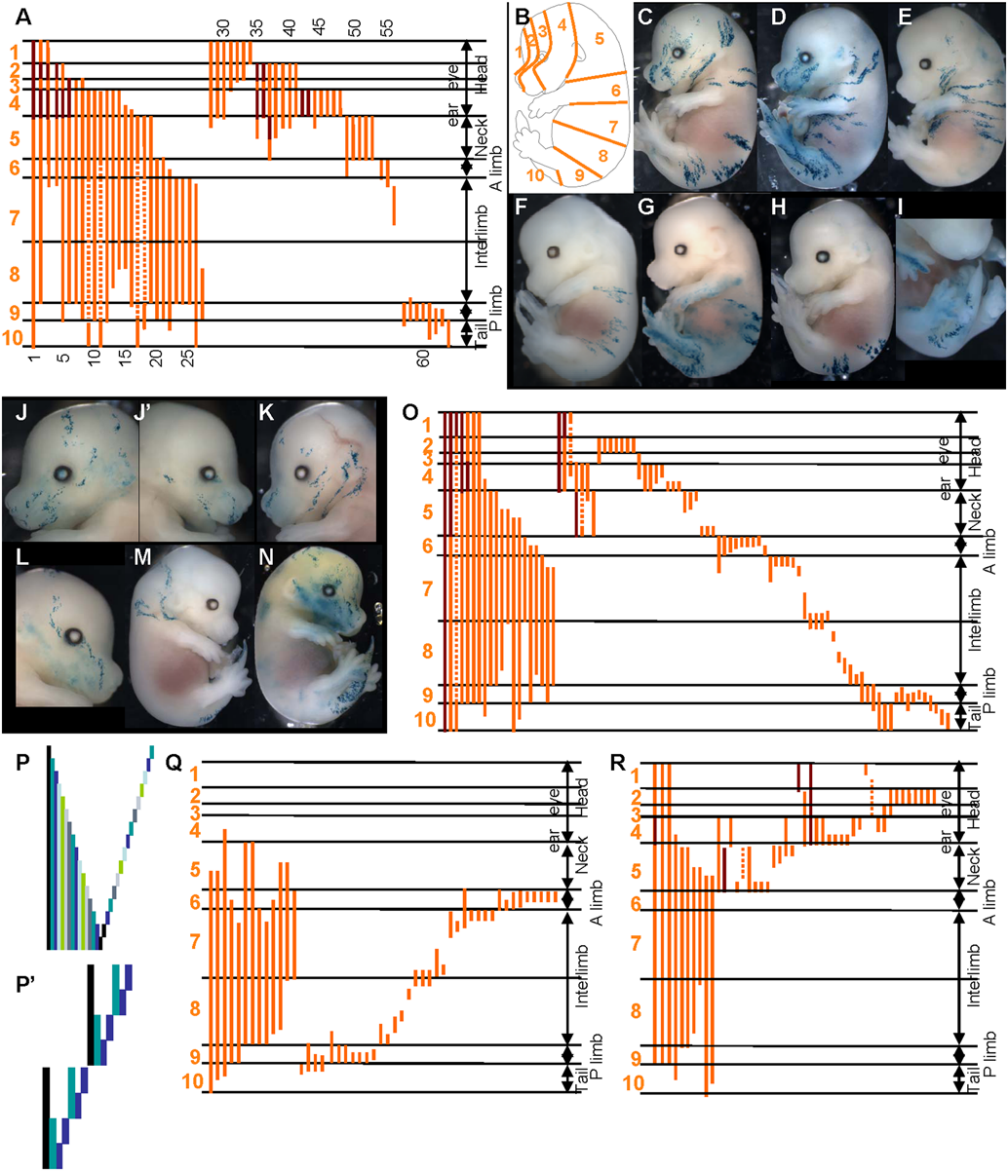
Three pools of SE-forming cells at E6-5-E7.5, following distinct modes of growth in the head and the trunk. (A) Schematic representation of the pattern of the 64 clones in E14.5 lox-LacZ embryos induced at E6.5–E7.5. Horizontal lines represent the boundaries between the regions of the body in A. Each vertical orange line corresponds to a single clone; no contribution to the levels where the line is interrupted. Dark red lines represent the contribution of the clone to the contralateral side. Clones were first classified according to size (long on left and short on right) and then according to the most anterior region to which they contribute. (B) Schematic representation of an E14.5 embryo showing the regions used in A. (C)–(M) Examples of E14.5 lox-LacZ embryos. (C)–(G) and (M) long clones; (H) and (I) posterior short clones; (J)–(L) anterior short clones. (N) is a spontaneous clone (in a CT2 embryo) labelled only in the head and the tail. (O) Schematic representation, as in fig. 4, of the pattern of the clones in E14.5 LaacZembryos. All clones were classified according to size (long on left and short on right) and then according to the most anterior region to which they contribute. (P), (P′) Pattern of clones expected from the labelling of cells in the pool of precursors classified according to the most posterior region to which they contribute for two representative models for their production: self-renewing pool of cells (P); and from the regional mode (P′). Each column (of different colour) represents a clone. (Q)–(R) Schematic representation, of the LaacZ clones that contribute (Q) to regions 6 to 9 (see B); (R) to regions 1 to 5 (see B). Clones were classified according to size (long on left and short on right) and the most posterior region to which they contribute. Note that clones from ancestral cells of the SE (shown in O) have been removed. (Q) Long clones that contribute to all regions are present on the left. (R) Clones from ancestral cells of head SE are shown on the left. Note the absence of clones that contribute to all five sub-regions.

To examine how the pool of founder cells produces the SE, we generated a lox-LacZ library of clones induced at E6.5 in [CT2×R26R] F1 embryos. Each of the 64 clones (Fig. 3A) had more than 200 cells. We estimate that the clones are born between E6.5 and E7.5, taking into account the delay and the time of action of 4-OHT [36] and the asynchrony of the embryos within and between the litters.

Three clones that contribute to structures from the head to the base of the tail (Fig. 3C–D) are similar to the LaacZ clones that correspond to the labelling of founder cells (Fig. 2K–K′). The SE founder cells are therefore still present in the embryo at E6.5.

The other clones contribute to the head and the trunk (Fig. 3E) or are restricted to a part of the AP axis: from the forelimb to the hindlimb (Fig. 3F), the posterior part of the trunk (Fig. 3G–I) or the head (Fig. 3J–L). These clones have no equivalent among the clones from the ancestral and founder cells of the SE; they have all the characteristics of sub-clones of the clones of founder cells, as each can be related to at least one of them (Fig. 3, compare E–H with Fig. 2K; G, J, K and L with C; I and J′ with D). This suggests that the initial pool of founder cells is becoming regionalized along the AP axis, defining a pool for the head (regions 1–5 Fig. 3B, clones 28 to 53, Fig. 3A), a pool for the trunk (regions 6–9 Fig. 3B, clone 20 to 27 and 54 to 56, Fig. 3A) and a pool restricted to the posterior regions (regions 9 and 10 Fig. 3B, clone 57 to 64, Fig. 3A). This posterior pool is unexpected as it is set aside at E6.5–E7.5 prior to the formation of the posterior regions of the embryo from E9.

The smallest lox-LacZ clones are composed of single DVCU in the head (Fig. 3L) but of groups of seven to 20 DVCUs in the trunk (Fig. 3F) and the tail (Fig. 3I). Therefore, some direct precursors of head DVCUs are present in E6.5–E7.5 embryos although only precursor cells of groups of trunk and tail DVCUs are present.

These results indicate: 1) The SE founder cells are present in E6.5 embryos. 2) This initial pool is rapidly regionalized into anterior, truncal and posterior regions. 3) In the anterior region, some of the direct precursors of DVCUs have already been produced although in the more posterior regions only precursors of large groups of DVCUS have been produced. This suggests that the production and individualization of the P-DVCUs progresses in a rostral to caudal direction.

### Head and trunk P-DVCUs are produced by regional and sequential modes respectively

P-DVCUs may be produced in the regions defined between E6.5–E7.5 in a regional mode or a sequential (self-renewing) mode. As the process of production proceeds in a rostral to caudal direction (see above), the modelling of the sequential mode of clonal growth for clones generated by random events (the LaacZ library) has been based on the functioning of a posterior pool. In a sequential mode, in which a posterior pool of precursor cells produces P-DVCUs during the establishment of the AP axis, long clones would be expected all contribute to the posterior pole of the embryo and clonal complexity (see terminology) would be expected to increase from anterior to posterior. Only small clones distributed homogenously along the axis of the embryo, and no intermediate-sized clones, would be expected (Fig. 3P). In a regional mode, the SE would form from the expansion of a few regions defined early. No clones contributing to the whole axis would be expected (the only long clones would be those derived from the ancestor cells) and clones would be intermediate-sized and smaller, and distributed homogenously along the AP axis. There would be no regions with greater clonal complexity than others (Fig. 3P′, in which the SE would be formed from two regions).

The distributions and the sizes of the clones of the LaacZ library (Fig. 30) were assessed and classified according to their most-posterior limit (Fig. 3Q, R) which corresponds to the most stringent condition for discriminating between the two models. The distribution of the clones in the region from the anterior part of the forelimb to the anterior part of the hindlimb (regions 6 to 9, Fig. 3B) was consistent with a sequential mode of growth (Fig. 3Q), this includes numerous long clones (Fig. 3Q, the 13 clones on the left) that contribute to the most posterior regions (regions 8 and 9) and many small clones distributed homogenously in the region. No intermediate-sized clone was observed. The distribution of clones in the head and neck (regions 1 to 5, Fig. 3B), however, was consistent with a regional mode (Fig. 3R): all clones were of intermediate size or smaller and were distributed homogenously in the region. All clones that contribute to all head-neck region result from labelling of the ancestor cells of this region (Fig. 3R, clones on the left).

These results suggest that two different modes of growth are used for the SE during AP elongation: a regional mode for the head-neck region and a sequential mode for the trunk, from a posterior pool. The boundaries between the regions, however, are not sharp. Some clones that contribute to the neck also contribute to the sequential production of the trunk (Fig. 3Q, clones at left) and clones that contribute to the trunk can also contribute to the tail (Fig. 3Q, 3 clones at left); the intermediate regions may be produced by a mixture of both modes of growth.

These observations do not describe completely the relationship between the P-DVCUs. This relationship is also dependent on dispersal properties of the cells in the regions considered.

### Precursors of DVCUs are dispersed along discrete longitudinal parasagittal lines along the AP axis

In order to analyse the dispersion of clonally related P-DVCUs during elongation, we determined the positions of the DVCUs of clones. A line was drawn, connecting the dorsal positions of the DVCUs of clones that have estimated birth dates between E6.5 and E7.5. We studied the 14 long LaacZ clones, the 27 most extensive clones in the E6.5 lox-LacZ library and 12 spontaneous clones in [CT2×R26R] embryos, all containing more than 400 cells. These clones will be referred to as the library of 53 clones.

**Figure 4 pone-0004353-g004:**
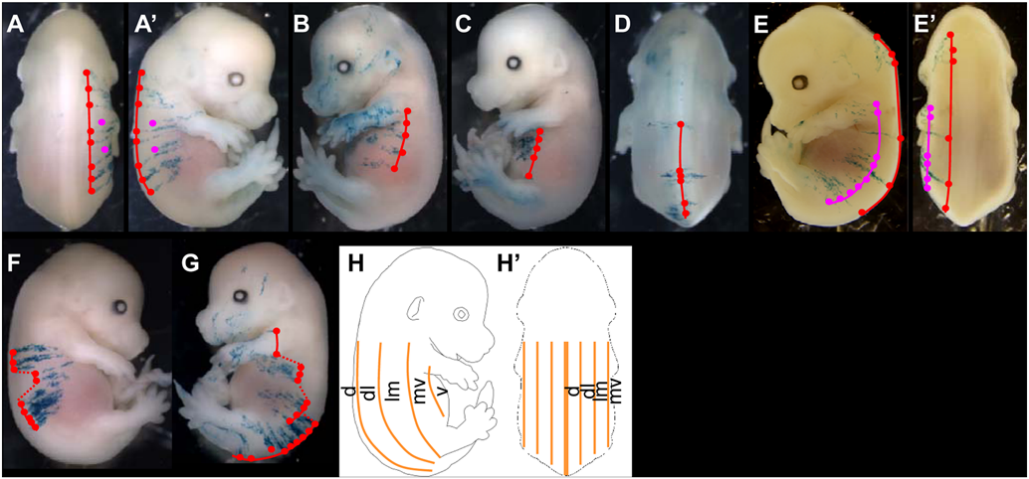
Cell arrangement of the clonally related P-DVCUs in the trunk. (A)–(G): Examples of clonal organisation in the trunk. The most dorsal positions of the DV-oriented stripes have been connected by one (A)–(D), two (E) or several (F), (G) lines, defining five discrete DV positions: dorsal (D), dorso-lateral (A), latero-medial (A, magenta line), mid-ventral (B), and ventral (C), schematically represented by yellow lines in (H and H′). A–A′, B and E–E′, from the LaacZ library; C, D, from the E6.5 lox- LacZ library; F, G: spontaneous labelling. A–A′ and E–E′ are two different views of the same embryo. d: dorsal, dl: dorsal-lateral, lm: lateral-medial, mv: mid-ventral, v: ventral.

For 27 of the 50 clones labelled in the trunk (regions 6 to 9, Fig. 3B), a single line traced parallel to the longitudinal axis of the embryo connected almost all DVCUs from the most anterior to the most posterior (n = 27/50, Fig. 4A–D). The DVCUs have therefore been produced by the same single cell in the posterior pool (defined in the paragraph above): this cell would have kept a constant DV position during the period of AP dispersion. For another 14 of these 50 clones, two lines traced parallel to the longitudinal axis of the embryo, were required to account for the observed labelling: a major line that starts at the most anterior position and a secondary line that starts more posteriorly (Fig. 4E, E′ red and magenta lines). It is likely that the cell at the origin of the secondary line was produced in the posterior pool by the cell at the origin of the major line (such that they are clonally related) and has shifted to a more ventral position in the posterior pool. Then the two cells in the posterior pool have kept a constant position during the production of the SE.

For another four of these 50 clones (Fig. 4F), the labelling shows groups of DV stripes spaced along the AP axis and shifted along the DV axis. The cell in the posterior pool probably moved during the production of the P-DVCUs. For the remaining 5 of the 50 clones, the labelling exhibits successive and ordered shifts along the DV axis (Fig. 4G); the cells of the clones show a strong tendency to shift from ventral to medial during AP dispersion (see below). In these last two cases, the shifts do not seem random and may correspond to defined DV regions of the embryo.

Thus, the labelling in the trunk reveals a clonal pattern, organised parallel to the AP axis of the embryo. The P-DVCUs produced by a single cell in the posterior pool contributes to the same longitudinal line. Shifts are observed, but only in a small number of clones and can be attributed to the behaviour of the cells in the posterior pool.

The shifts (Fig. 4E–G) suggest longitudinal organization and also organization in a defined dorso-ventral sector. We analyzed the DV positions adopted during AP dispersal of the 56 long lines of the clones with one or two longitudinal lines. The lines are found in only five DV positions (Fig. 4H, H′). Twenty-four lines are in a dorsal position, lateral to the dorsal midline of the embryo (Fig. 4A). This position dorsally limits a dorso-lateral region. Eleven lines are in a more lateral position that delimits a latero-medial region (Fig. 4E, magenta line). Nine lines characterize a more lateral position, the stripes being ventral to the limbs, limiting dorsally a mid-ventral region (Fig. 4B). Five lines characterize a fourth position (Fig. 4C), referred to as ventral, that limits dorsally a region that reaches the ventral midline. Seven lines characterize the last position: the middle of their DV stripes approximately coincides with the midline of the embryo (Fig. 4D). This position is referred to as dorsal. Therefore the trunk is characterized by only five longitudinal sectors.

For the clones exhibiting two lines or shifts, the lines were always in adjacent positions, for instance dorso-lateral and latero-medial (Fig. 4E), or mid-ventral, latero-median and dorso-lateral (Fig. 4G). This applies to almost all “shifted” clones (n = 21/23; 41/44 shifts).

**Figure 5:**
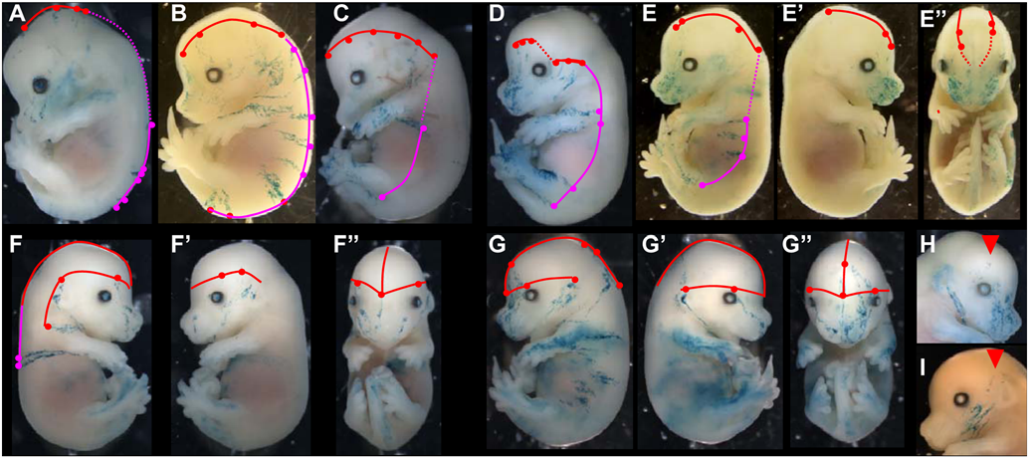
Cell arrangement of the clonally related P-DVCUs in the head. (A)–(G): Examples of clonal organisation in the head. The most dorsal positions of the DV-oriented stripes have been connected by one (A)–(C), two (D), (E) or three (F)–(G′′) lines, defining four discrete DV positions: dorsal (A), dorso-lateral (B),(E), latero-medial (C) and mid-ventral (D), (F), (G). Points indicate the most dorsal position of the DVCU. The red lines connect DVCUS in the head, the magenta lines connect DVCUS in the trunk. (H), (I): Ventral labelling in the head. These labellings are connected with more dorsal head regions (arrowheads) but not with the ventral trunk region. (A), (F), (G) from the E6.5 lox-LacZ library; (B)–(E), (H)–(I): from the LaacZ library (F, F′, F′′; G, G′, G′′ and E, E′, E′′ different views of the same embryo).

In the head (regions 1 to 5, Fig. 3B), among the 15 clones (of the 25 that contribute to the head in the library of 53 clones) that have an extension sufficiently long to define the line that connects the dorsal limit of their DVCUs, 12 have only one line. The line is, as in the trunk, parallel to the longitudinal axis of the embryo (n = 12/15, Fig. 5A–C red lines). Two of the other three clones show two lines parallel to the AP axis and the last one shows a shift (Fig. 5D red line). The AP dispersion therefore respects the relative DV position of the clonally related DVCU in all cases except one, and, as in the trunk, the organization is longitudinal and parallel to the embryonic AP axis.

Nine of these 15 clones show bilateral labelling in the most rostral part of the head (Fig. 5E–E”). In most cases (n = 5/9), the lines are at the same DV level on either side of the embryo, dorso-lateral (Fig. 5E–E′) or latero-ventral. Similar labelling was observed in clones that contribute only to the head. This suggests that the dispersion of the cells at the origin of the left and right contribution follows an identical line and continues unchanged rostrally (Fig. 5E–E′). The most complex clones in the head can be resolved by adding one further line that generally links very dorsal DVCUs (Fig. 5F–G′′, n = 3).

We examined clonal continuity between the head and the trunk. Among the 15 clones in the head, 9 extend to the trunk without change in the DV position of their DVCUs (Fig. 5A, B, D, F and G; magenta lines). The other six exhibit a shift at the neck level (Fig. 5C). Six of the 10 clones that have a short extension in the head also extend to the trunk, without change in their most DV position. Thus, 15 of the 25 clones show continuity of the most dorsal position of their DVCUs from the head to the trunk and therefore the regions defined in the trunk have a correspondence in the head. This applies to the dorso-lateral (Fig. 5B), the mid-ventral (Fig. 5D) and the dorsal lines (Fig. 5A, F G). However, the ventral region cannot be defined this way in the head: the ventral clones are genealogically closest to cells from more dorsal regions in the head (Fig. 5H–I, arrowheads).

Note that this correspondence between the trunk and the head only concerns the step of AP dispersal of P-DVCU, not their contribution to the different DV regions. Although the trunk DVCUs are generally restricted to one (Figs. 5E, 3F) or two (Fig. 4E, E′) adjacent regions, this is not true for the head: most DVCUs contribute to several regions lateral to their most dorsal position, frequently as far as the most ventral head domain (Figs. 5H, 3J–L).

This study reveals a general organization of the P-DVCUs in five sectors in the trunk and four positions in the head. These sectors that lie parallel to the main axis of the embryo, are respected during the AP dispersal of the P-DVCUs. These characteristics are observed whatever the mode of production of the P-DVCUs (sequential in the trunk and regional in the head). The dorso-ventral expansion of these longitudinal sectors forms the next DV regions in the SE. This DV expansion follows different rules in the head and trunk.

In addition to this organization in longitudinal sectors, the clonally related DVCUs of a single sector are spatially separated along the AP axis; therefore we investigated the origin and uniformity of the spacing.

### Cell intercalation in the longitudinal sectors

As the P-DVCUs are organized longitudinally, clonally related cells are expected to form continuous clonal columns in absence of cell rearrangement, irrespective of their mode of production. However this is not observed (Figs. 3–5). We studied the spacing of the DVCUs in clones in detail to assess the extent of the cell rearrangements.

**Figure 6:**
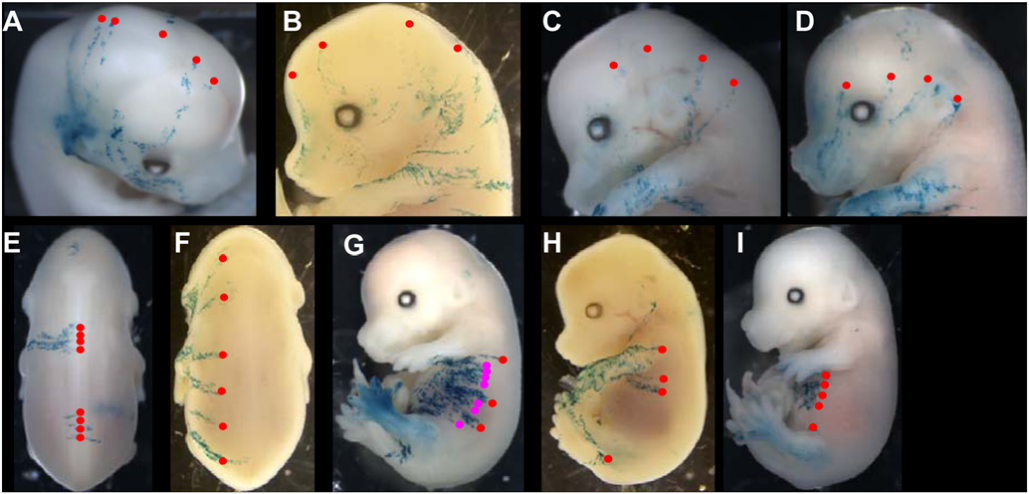
Spacing of the clonally related P-DVCUs. (A)–(D): Examples of spacing in the head for the four DV positions: (A), dorsal, (B), dorso-lateral; (C), latero-medial and (D) medial. In D the magenta point indicates a second dorsal line of dispersion. (E)–(I): Examples of spacing in the trunk for the five DV positions dorsal (E), dorso-lateral (F); latero-medial (G, red points); medial (G, magenta points and H); and ventral (I). Note that the spacing is greater dorsally than ventrally. (A), (E), (G), (I) from the E6.5 lox-LacZ library; (B)–(D), (F): from the LaacZ library; B and F are two views of the same embryo; (H): spontaneous labelling. The points represent the most dorsal position of the DVCUs.

In all trunk DV regions and at all DV levels in the head, the clonally related DVCUs are well separated from one another (Fig. 6), evidence of cell rearrangement leading to cell intercalation of the P-DVCUs whether in the dorsal (Fig. 6A–E), dorso-lateral (Fig. 6B–F), latero-medial (Fig. 6C–G) and mid-ventral (Fig. 6D–H) regions for the head and trunk, and in the ventral (Fig. 6I) region for the trunk. The spacing is variable in all regions (Fig. 6. compare G to H) but the ventral region of the trunk usually exhibits moderate or no spacing (Fig. 6I, n = 4/5).

Consequently, cells of different clonal origins intercalate to form a given sector. This intercalation takes place in a context of regional production in the head and of sequential production of the P-DVCUS in the trunk. Intercalation is advanced at E7.5 in the head, but is not finished and continues after this stage in the trunk. A more pronounced intercalation is observed in the dorsal and dorso-lateral than ventral regions of the trunk (Fig. 6. compare E, F to G, I), possibly indicating a lateral to medial direction of the cell rearrangement.

### Relationship between the head, trunk and tail pools of founder cells

Finally, to determine the relationship between the pools of founder cells of the three AP regions, that is to understand how the trunk and tail pools of precursor cells are formed, we searched for clonal continuity between these three regions.

#### Formation of the trunk pool

Eighteen of the 64 clones induced at E6.5 (lox-LacZ library) contribute to both the head and the trunk (region 1–5 and 6–9 respectively, Fig. 3B, C–E; Fig. 5A, F, G). Therefore there are still common precursors at E6.5–E7.5 for both structures on which the head-trunk clonal continuity is built.

In ancestral clones from the LaacZ library, no clones that contribute to the trunk but not to the head were found (n = 0/7). Therefore, all clones that contribute to the trunk also contribute to the head (Fig. 2A–G, K), indicating that perhaps all of the trunk pool cells are derived from precursor cells common to the head and the trunk.

**Figure 7:**
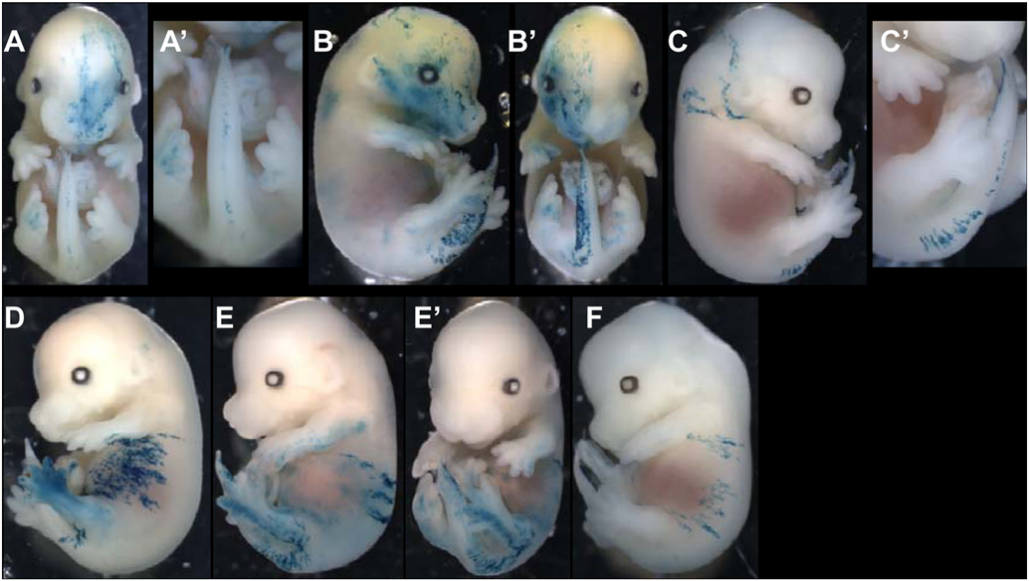
Relationship between the pools of founder cells. (A)–(C′): examples of clones whose contribution is restricted to the head and the tail. (D)–(E′): examples of clones that contribute to both the trunk and posterior regions. (F): an example of a clone that stops at the level of the hindlimb. (A–A′, D, E–E′, F) from the E6.5 lox-LacZ library; (B–B′): spontaneous labelling; (C–C′) from the LaacZ library.

The library of 53 clones (with estimated date birth between E6.5 and E7.5) was searched for clones contributing to the head but not to the trunk, to determine if all founder cells of the head SE contribute to the trunk pool. Six such clones were found: they participate in the head and the tail and/or the posterior part of the trunk, but not to the region between the forelimb and the hindlimb (Fig. 7A–C′). These clones reveal the existence of head SE founder cells that do not contribute to the trunk pool.

The clones, which show several lines of dispersion in the head, provide additional information. Among the bilateral clones in the head, one line of dispersal always ends in the region between the external ear and the forelimb (n = 11/11, Fig. 5E′). In the most complex labelling in the head SE, with three lines of dispersal (n = 3/9), one (Fig. 5F′) or even both (Fig. 5G, G′) lines on the sides stop, again in the region between the ear and the forelimb. This shows that precursor cells, common to the head and the trunk, produce descendant cells that contribute only to the head (represented by clones with the lines that do not extend to the trunk). Most lines (n = 17/28) that extend from the head to the trunk are dorsal or dorso-lateral (Fig. 5A, B, E, F, G). Moreover, the lines that extend from the head to the trunk appear frequently to shift their position at the level of the neck. The shift is in all cases directed ventrally (n = 17/17, Fig. 5C), sometimes with a shift of more than one adjacent position, cells shifting from dorso-lateral to mid-ventral, for example (Fig. 5E).

Therefore the trunk pool is formed of cells that have common ancestors with the cells that constitute the head SE, including its most anterior part. These precursor cells form a common head-trunk pool at E6.5–E7.5 that constitutes the main and possibly only clonal origin of the trunk pool. It also generates precursor cells that contribute only to the head SE. It is mainly the cells that have a dorsal or dorso-lateral position in the head that form the trunk pool. The lateral regions in the trunk are in part formed by ventrally shifted cells that are more dorsal in the head.

#### Formation of the posterior pool

The most posterior part of the trunk is produced by a restricted pool of cells set aside between E6.5 and E7.5 (lox-LacZ library: Fig. 3A, clones 61 to 64, Fig. 3I). Six non-restricted clones found in the lox-LacZ and the LaacZ libraries exhibit labelling only in the head and the tail (Fig. 7A–C′). It suggests that at least part of the posterior pool is derived from the regionalization of founder cells (represented by these six clones) whose participation is restricted to the head and the tail.

The trunk pool distributes clones up to the posterior limit of the hindlimb, or beyond (Fig. 3A). We searched the library of 53 clones for examples contributing to both the trunk and these posterior regions, and four such clones were found. Two contribute only to the proximal part of the tail (Fig. 7D) and the other two, to both the proximal and distal parts of the tail (Fig. 7E–E′). The other four clones in the library that participate in the same region of the trunk all stop at the level of the hindlimb (Fig. 7F). Thus, the pool of cells that participate in the anterior trunk can also contribute to the posterior trunk (4 out of 9), but rarely to the most distal part of the tail. Very similar clones were also found in the LaacZ library.

In conclusion, the posterior pool is formed partly by cells from the trunk pool and partly by a pool set aside at E6.5–E7.5 of which at least a part is derived from the regionalization of SE founder cells restricted to the head and the tail.

## Discussion

### The combination of two complementary methods of cell labelling

We combined two methods of cell labelling to provide a particularly powerful tool for describing cellular aspects of morphogenesis.

The LaacZ system has been rendered ubiquitous by introducing the reporter gene into the ROSA26 locus [24]. The ROSA26LaacZ method permits visualization of all cells of clones, including those outside the structure of interest; it also ensures that no territory is excluded from the analysis. Our study validates this approach, so that it can now be used to study more complex systems or intricate periods of development such as gastrulation.

A system of production of labelling in which the birth date is controlled (GICL) [20] has also been made ubiquitous by using ROSA26-driven Cre recombinases. We used this system to generate polyclonal labelling and clonal labelling induced during gastrulation. The ability to generate polyclonal labelling allowed clonal saturation and therefore testing of the generality of the conclusions for any given structure. Polyclonal labelling can readily be generated using lines that present a moderate background of spontaneous recombination, as illustrated here with ROSA26cre-ER^T^ [33]. The ability to generate clonal labelling allows saturating critical periods of development. Clonal labelling requires a Cre recombinase that presents a low frequency of spontaneous labelling such as the CT2 line. The CT2 line made possible the generation of a saturated E6.5 lox-LacZ library, in a way that would have been impossible with the LaacZ system. It also allowed classification of the LaacZ clones generated in the period before, during and after the period covered by the lox-LacZ library. By saturating at E6.5 we could validate two families of clones, those that participate only in the head and the posterior part of the embryo and those that derive from cells set aside early in development that contribute to the formation of the posterior part of the embryo only. This observation is critical to interpret SE morphogenesis. However, the general use (that is not restricted to early stages) of the temporal system of clonal labelling is limited by the spontaneous generation of labelling ([37], [38]; this article). Nonetheless, the power of this approach is now demonstrated ([20]; this article), so it would be beneficial to find ways to abolish the spontaneous generation of labelling, to make it more generally applicable.

### Sophisticated modes of SE formation

Morphogenesis of epithelial tissues generally involves an isotropic mode of cell proliferation [35], [39]. This mode does not require either cell movement or cell orientation or other sophisticated cell behaviour [13]; it engages only minimal cellular operations and genetic control. Therefore, it would seem particularly adapted to the growth of the SE whose main known property is to cover the embryo. Indeed, it is this simple mode of growth that is observed in the SE after E14, even if the final size and shape have not been attained. However, before E14, the SE does not grow in an isotropic mode. Between E14 and E9, growth is in dorso-ventrally oriented stripes and from E6.5 to about E9 growth is dispersive, along lines parallel to the longitudinal axis of the embryo with clonally related cells interspaced. It is the completion of the spacing of the P-DVCUs that defines the transition between the period of growth of the DVCUS and the period characterized by these lines. The succession of two sophisticated modes of growth demonstrates that SE formation is tightly organized and controlled. It suggests that these characteristics may have developmental origins.

### SE elongation by both cell rearrangement and cell proliferation

#### Spacing and mediolateral cell intercalation

The spacing of the clonally related P-DVCUs is characteristic of dispersive growth during the first phase of SE formation. Since, in addition, P-DVCUs are organized into a small number of sectors (manifest as the few discrete DV positions possible for the lines along which P-DVCUs are spaced), this implies a special cell rearrangement process. In the absence of cell rearrangement, clonally related cells would remain close, in columns or stripes, as they do later in the DV stripes and as other cells do in other systems (the clonal columns in the cortical structures of the CNS [40], [41] and the longitudinal columns of the tubular structure of the kidney [42], [43]. Visibly, in the SE, this is not the case: each sector has a polyclonal origin and the cells of the polyclone rearrange.

For the rearrangement to produce lines that are parallel to the axis of the embryo the underlying process must be ordered. It must separate daughter cells without changing their relative DV position. As this ordered process involves several adjacent sectors, it must act throughout the DV dimension of the structure. In the trunk and the tail the spacing is stronger dorsally than ventrally, suggesting that the process involved is oriented towards the midline.

An obvious consequence of this spacing is elongation of the head, trunk and tail SE. In lower vertebrates AP extension is due to convergent extension [2], [44], [45], [46] by medio-lateral intercalation during gastrulation. Examples include the zebrafish [1], [47] and Xenopus [48], in which convergent extension has many similarities with the process described above: most importantly, it respects the medio-lateral order of cells [47]. In the mouse convergent extension has been proposed to play a role in the developing neural plate [49], [50]. So the most obvious explanation for the spacing of the clonally related P-DVCUs of the SE, their organization in lines parallel to the AP axis and the DV sectorisation, is convergent extension, a hypothesis that can now be tested.

Several observations suggest that the process at the origin of the spacing of P-DVCUS follows a spatio-temporal progression from anterior to posterior. The smallest clones induced at E6.5–E7.5 in the head have only one or two P-DVCUs although those in the trunk or the tail have numerous P-DVCUs indicating that the spacing stopped in the head at a stage when it is still operating in the trunk and the tail. This suggests a caudally oriented progression of the initiation of the spacing.

We conclude that the spacing of the PUCDVs is very likely the consequence of medio-lateral convergent extension of the SE. As the SE of the posterior territories (the tail) is organized into sectors and shows similar spacing, convergent extension continue even after the regression of the primitive streak and the closure of the neuropore.

#### Combination of distinct modes of cell proliferation with spacing

The analysis of the mode of growth of the cells producing the P-DVCUs suggests a sequential mode in the trunk: production from a pool of caudally positioned cells that self renews during the process and produces the P-DVCUs anteriorly. The caudally polarized clones that start in the middle of the trunk, in the E6.5 lox-LacZ library, indicate that this system produces the P-DVCUs for the anterior trunk by E7.5. However, as no clones corresponding to only one P-DVCU were found in the anterior trunk, spacing is not yet started. Consequently production and spacing of the P-DVCUs are likely two separate and sequential operations. Both progress from anterior to posterior.

In the head, the production of the P-DVCUs is regional. If the spacing of the cells in the head uses the same cellular operations as in the trunk these operations could be combined with different modes of division, suggesting a modular organisation [51], [52] of these operations. Hair follicle morphogenesis is another system for which a separation of growth and cell rearrangement has been observed [20].

### A reorganisation of the pool of producers of P-DVCUs at the rhombencephalon level

The organization of clonally related DVCUs in certain DV positions suggests DV organization in the pool of P-DVCU producer cells. This DV organization is established very early: clones from the ancestral and founder cells of SE already exhibit a DV restriction. This organization is maintained during the elongation of the head and of the trunk, as demonstrated by most clones having only one line parallel to the AP axis of the embryo in each of these structures. This striking observation shows that the process of elongation is not accompanied by random cell mixing along the DV axis, but respects some sort of coherence. This coherence could be part of the process of oriented AP elongation and/or preliminary DV organization.

Many lines continue unchanged in the head and the trunk and in particular in the more dorsal regions, revealing a continuity of properties. This is accompanied by a clonal continuity between the pools of cells that produce the head and the trunk: indeed, the pool that produces the trunk P-DVCUs is probably entirely derived from cells that have produced head P-DVCUs.

Nevertheless neither the clonal nor the cellular organization is totally conserved. First, not all cells of the head contribute to the trunk pool, but mainly the dorsal and dorso-lateral ones. Second, many of these cells move ventrally (the so called shifts). These indicate that there is a transition involving cell rearrangement and the acquisition of novel properties (such as a change in the fluidity of the tissue). This transition requiring a change in the developmental programmes of the cells occurs at the level of the rhombencephalon, that is, before the production of the anterior trunk. It may be as early as the initiation of gastrulation, raising the possibility that the trunk pool is organized by signals from the anterior primitive streak. The transition may accompany the individualization of the trunk organizer from a structure that contains the anterior organizer [8] and controls early SE development in the head. This would require novel programming of the precursor cells of the SE (acquisition of a novel mode of growth and for a short period of time, novel dispersal behaviour). It should be noted that the transition occurs in a context of a closed SE pool: all cells that contribute to the trunk SE are cells that have previously produced SE. Clearly, clonal continuity in this case does not mean continuity of cell properties. The precursor cells of a structure can change.

Whether these changes occur in concert with the evolution of other long-term axial progenitors [14], [16], [53], [54], [55], [56], [57] in particular neural progenitors with which SE progenitors share many properties, can now be experimentally tested.

### Dorso-ventrally oriented stripes

The DV oriented stripes of cells are a general characteristic of the growth of the head, the trunk and the tail SE. This type of oriented growth implies a particular behaviour: either oriented cell division [13], [58] and/or realignment of daughter cells after mitosis [59]. In both cases, this requires planar polarization of the epithelium, suggesting that the planar cell polarity pathway [60], [61] may be involved. This planar polarity of the whole epithelium must be maintained, until the transition to the isotropic mode of growth.

### Head-tail clones, vestiges of the radial to bilateral transformation in bilaterians?

The single clonal origin of the precursor cells for the trunk SE contrasts with the double clonal origin of the precursor cells for the head SE and for the SE of the posterior part of the embryo. Indeed, some head founder cells and some tail founder cells do not give rise to, or are not derived, from trunk founder cells: they are head and/or tail specific. Surprisingly, these two specific pools have (at least in part) the same clonal origin as evidenced by clones that contribute only to the head and the tail. A pool of common cells for anterior and posterior SE is therefore present early. This pool may contribute to as much as half of the founders of head and tail SE.

Considering the origin of the axial tissues in bilaterians [62], [63], [64] perhaps helps explain this observation. In the common ancestor of deuterostomes and maybe even in the common ancestor of both deuterostomes and protostomes [65], the closure of the blastopore involves movement of lateral cells that separate the future anterior region (where the stomodeum will form) from the future posterior regions (where the anus will form). This cell movement closing the blastopore facilitates the radial to bilateral transformation of the embryos [66] and the appearance of the dorsal neural plate in deuterostomes and of the ventral neural cord in protostomes. We propose that this movement has been conserved in vertebrates and that it may have facilitated a new mode of cell proliferation (the sequential mode) for trunk elongation in an intense cell proliferation context. The clones restricted to the head and tail SE, the existence of three pools of cells at the origin of the longitudinal organization of the SE and the mediolateral convergent extension of the SE in the mouse may be the consequence of this founder episode.

## Materials and Methods

### Transgenic mouse lines

The R26LaacZ1.1 line (from Elena Tzouanacou and Valerie Wilson) was obtained by introducing a *LaacZ* reporter gene by homologous recombination (HR) into the ROSA26 locus; the size of the duplication is 1117 bps. The CT2 line (from Lars Grotewold and Austin Smith) was obtained by introducing the *CreER^T2^* gene [31] by HR into the ROSA26 locus. The ROSA26cre-ER^T^ line was from Anton Berns [33] and the R26R Cre reporter mouse from Philippe Soriano [26]. In this line, a loxP-flanked-stop-sequence (PGKneo-polyA) upstream from the *LacZ* gene was introduced into the ROSA26 locus by HR. The ROSA26 promoter confers ubiquitous expression on LacZ, CreER^T^ or CreER^T2^.

### Generation and observation of embryos

[R26R×ROSA26CreER^T2^] and [R26R×ROSA26CreER^T^] embryos were obtained by crosses between CT2 or ROSA26cre-ER^T^ males and superovulated R26R females. LaacZ embryos were obtained by crosses between homozygous R26LaacZ1.1 males and C57Bl/6 or Swiss superovulated females. Embryos were staged with the day after crossing being defined as embryonic day of development (E) 0.5. Embryos were dissected in PBS, fixed by incubation in 4% paraformaldehyde for 30 minutes, rinsed twice in PBS, stained in X-gal solution (4 mM K_3_Fe(CN)_6_, 4 mM K_4_Fe(CN)_6_, 2 mM MgCl_2_, 0.5 mg.mL^−1^ X-Gal in PBS) at 37°C for 48 hours to reveal β-galactosidase activity, rinsed twice in PBS and postfixed by incubation in 1% paraformaldehyde.

### 4-OHT preparation and injection

Initially, 4-OHT was prepared in a hydrophobic solvent and injected intraperitoneally (ip) as in [67]. 4-OHT was suspended at a concentration of 100 mg.mL^−1^ in 100% ethanol, diluted in autoclaved corn oil to 10 mg.mL^−1^, sonicated for 30 minutes and stored at −20°C. Before injection, the suspension was diluted in corn oil to the desired concentration and vortexed. This protocol was used to prepare 4-OHT for polyclonal labelling in [R26R×ROSA26CreER^T^] embryos using 66 µg.g^−1^ (ip). Subsequently, we used a novel protocol [36] involving cremophor® EL (Sigma) and intravenous (iv) injection. 4-OHT was diluted to 20 mg.mL^−1^ in 100% ethanol, then diluted in cremophor® EL (Sigma) to 10 mg.mL^−1^, and again in 1X PBS to 1 mg.mL^−1^. Before injection, the suspension was diluted in 1X PBS to the desired concentration. Labelling was initiated by the intravenous injection (into the vein of the tail) of 4-OHT into pregnant mice at various times after coitus. This protocol was used to prepare 4-OHT for clonal labeling at E6.5 in [R26R×CT2] embryos using 0,44 and 0,33 µg.g^−1^ (iv) (Table 3).

### Statistical analysis of the LaacZ library of clones

The labeling of a cell by spontaneous intragenic homologous recombination within the LaacZ gene is a random event [11]. The frequency of N independent recombination events can be calculated by the fluctuation test of Luria-Delbrück [68]. The expected number of embryos with N independent recombination events is N0 (ln (Ne/N0))^N^/(N!), where N0 is the number of embryos observed with no recombination event (N = 0) and Ne the total number of embryos of the sample. Table 1 gives the calculated number of embryos with N events of recombination for a sample of 97 embryos of the LaacZ library and the observed number of embryos with possibly N recombination events.

The frequency of events corresponding to the combination of two labelings of two different categories, A and B, equals the product of the probability of each single event. That is C = NA×NB/(Ne)^2^, where NA is the number of observations of event A, NB of event B, and Ne the total number of embryos in the library. Table 2 gives the number of events for each size category for the sample of 97 embryos and for the 4248 embryos of the entire library.

### Statistical analysis of the lox-LacZ library of clones induced at E6.5

The clonality of a labeling is documented by statistical tests. For a SE library of E6.5 lox-LacZ clones observed at E14.5 the expected size of the clones induced is 256 cells (that is, 2^8^, assuming a doubling time of 24 h) or more (assuming a doubling time shorter than 24 h). In preliminary experiments we tested and confirmed the hypothesis that the number of clones in these categories (and not the others) is effectively increased. The E6.5 lox-LacZ library has been produced using concentrations of 4-OHT (0.33 to 0.44 µg.g^−1^) that give a frequency of labelled embryos between 1 and 2×10^−1^ (Table 3). In this condition, the probability of two independent events is 4×10^−2^ (according to the formula C = NA×NB/(Ne)^2^, where NA = NB = 2×10^−1^, see above) which is practically negligible and possibly identifiable (the second event being potentially anywhere in the embryo including in the contralateral side to the first labeling).

To validate the library, spontaneous labeling of the category of clones to be induced must be substantially lower than 2×10^−1^. The frequency of spontaneous labeling of clones with 200 to 400 cells was actually only 1.3×10^−2^ (2/144) and that of clones with more than 400 cells was 2×10^−2^ (3/144) (Table 3, first line, non-injected controls).

To assess a possible variability within the pool of induced embryos, Fisher's exact test was used to compare the labeling between litters (2 by 2) and the two extreme categories (the least labelled potentially not induced and the most labelled potentially too induced) were discarded. The statistical analysis, including the degrees of confidence (from p = 0.019 to p<0.0001), of the final library (64 labeling among 287 embryos) that confirms its validity is reported in Table 3.
